# Prenatal Irradiation-Induced Hippocampal Abnormalities in Rats Evaluated Using Manganese-Enhanced MRI

**DOI:** 10.3389/fncir.2018.00112

**Published:** 2018-12-17

**Authors:** Shigeyoshi Saito, Kazuhiko Sawada, Ichio Aoki

**Affiliations:** ^1^Division of Health Sciences, Department of Medical Physics and Engineering, Graduate School of Medicine, Osaka University, Osaka, Japan; ^2^Department of Nutrition, Faculty of Medical and Health Sciences, Tsukuba International University, Tsuchiura, Japan; ^3^Group of Quantum-State Controlled MRI, National Institutes for Quantum and Radiological Science and Technology, Chiba, Japan; ^4^National Institute of Radiological Sciences, National Institutes for Quantum and Radiological Science and Technology, Chiba, Japan

**Keywords:** radiation injury, radiation-induced abnormalities, mossy fiber distribution, hippocampus, manganese-enhanced MRI

## Abstract

The aim of this study was to characterize hippocampal abnormalities in rats after prenatal x-ray irradiation using manganese-enhanced MRI (MEMRI). All radiation-exposed rat brains showed a reduced volume with prominent dilatation of lateral ventricles. Moreover, MEMRI-enhanced areas within the hippocampus were reduced in volumes by approximately 25% of controls, although the entire volume of hippocampus was decreased by approximately 50% of controls. MEMRI signals were enhanced strongly in the hilus and granular layer of the dentate gyrus (DG) and the pyramidal layer and infrapyramidal region of the CA3 region, and moderately along the CA1/2 pyramidal cell layer in the control rats. In radiation-exposed rats, MEMRI signals in the CA1/2 regions disappeared due to disrupting their laminar organization, although strong MEMRI signals were sustained in the DG and CA3 regions. Histopathological examinations in radiation-exposed rats revealed disorganizations of the DG granule cell layer and the CA3 pyramidal cell layer with reducing the cell density. The CA1/2 pyramidal cell layer was disrupted by invading ectopic cell mass. Neural cell adhesion molecule (NCAM)-positive fiber bundles were sustained in radiation-exposed rats, although they distributed aberrantly in the suprapyramidal CA3 region with a slight reduction of NCAM staining. Furthermore, glial components consisted largely by astrocytes and minor by microglia were densely distributed in the DG rather than in other hippocampal regions, and their density radiation-exposed rats. In conclusion, MEMRI signal enhancements could delineate different neuronal and/or glial components among hippocampal regions. We characterized microstructures of the deformed hippocampus as well as its macrostructures in a prenatally radiation-exposed rat model using *in vivo* MEMRI. The present findings provide advantageous information for detecting nondestructively hippocampal deformations in neurodevelopmental disorders.

## Introduction

Radiation exposure to embryos/fetuses can cause severe problems of the central nervous system such as hydrocephalus and microcephaly (Kameyama, 1987; Otake and Schull, 1998). Miki et al. (1999) revealed histological alterations in the rat hippocampus after prenatal x-ray irradiation and their severity increasing in a dose-dependent manner (Miki et al., 1999). They also showed involvements of dilatation of lateral ventricles and a disruption of vascular endothelial cells in irradiation-induced hydrocephalus (Miki et al., 1999). Takai et al. (2004) reported a relationship between cognitions and hippocampal structural changes after prenatal radiation exposure (Takai et al., 2004). Rats exposed prenatally to gamma-rays also showed a growth retardation and behavioral alterations that persisted postnatally and throughout life (Wang et al., 2005). Those previous studies evaluated effects of prenatal exposure to radiation in rodents using histopathological and behavioral approaches (Miki et al., 1999; Takai et al., 2004; Wang et al., 2005). There have been no previous reports that have characterized cellular and morphological alterations in the hippocampus of prenatal radiation-exposed rats by *in vivo* MRI.

Manganese-enhanced MRI (MEMRI) can detect changes in functional connectivity in the brain at a high resolution *in vivo* (Aoki et al., 1999). Manganese chloride alters T_1_ values so that a signal enhancement is detectable with T_1_-weighted MRI at approximately 1 mM local tissue concentration or less (Aoki et al., 2004; Silva et al., 2004). For example, MEMRI can clearly distinguish laminar structures of hippocampal formation (Hf) and cerebral cortex *in vivo* (Silva et al., 2004). There are several hypotheses for the mechanism of manganese enhancement in brain disorders such as astrogliosis (Kawai et al., 2005, 2007), hippocampal mossy-fiber sprouting (Nairismägi et al., 2006), and manganese super oxide dismutase (Mn-SOD) expression (Yang and Wu, 2007; Immonen et al., 2008). The goal of the present study was to characterize the hippocampal microstructures using a prenatally x-ray irradiated rat model using *in vivo* MEMRI. Histological and immunohistochemical examinations were also performed in parallel for identifying the *in vivo* MEMRI detectable microstructures. The present results will provide valuable insights for investigating nondestructively brain deformations using living rodent models of neurodevelopmental disorders.

## Materials and Methods

The Animal Welfare Committee of the National Institute of Radiological Sciences (NIRS) approved this study. Pregnant Sprague-Dawley rats (*n* = 6, 250–280 g, Japan SLC, Hamamatsu, Japan) were allowed to rest for 1 week before the experiment. The animals had free access to food and water, and were kept under standard laboratory conditions with a room temperature 22–23°C, approximately 50% humidity, and a 12 h light/dark cycle. Six pregnant rats were divided into two groups consisting of non-radiation-exposed control (*n* = 3) and radiation-exposed (*n* = 3) animals. The radiation-exposed group underwent a single exposure to whole-body x-ray irradiation at a dose of 1.5 Gy on day 15 of pregnancy. Based on a previous report (Takai et al., 2004), x-ray irradiation conditions were 200 kVp, 20 mA, 0.5 mm Cu +0.5 mm Al filter, 110 cm distance from focus to object and 0.27–0.28 Gy/min dose rate. After birth, 10 female rats were selected at random from both control rats (*n* = 5) and radiation-exposed rats (*n* = 5). The body weight was 44.4 ± 0.98 g in radiation-exposed rats at 3 weeks of age, significantly smaller than that for age-matched controls (49.6 ± 1.31 g; *p* < 0.001). All female neonatal rats, regardless of being control or radiation-exposed, were kept with the mother rats in regular light/dark cycles until the MRI experiments were performed at 3 weeks after birth.

All MRI experiments were performed on a 7.0 T MRI scanner (Magnet: Kobelco and JASTEC Japan; Console: Bruker Biospin, Germany) with a volume coil for transmission (Bruker) and 2-channel phased-array coil for reception (Rapid Biomedical, Rimpar, Germany). MnCl_2_ was used for assessment of neuronal activity or ventricle dilatation. Immediately prior to and during the MRI scan, all rats were anesthetized with 2% isoflurane (Abbott Japan, Japan). To maintain appropriate body temperature during the scan, rats were placed on a cradle with a heater. Rectal temperature was continuously monitored and maintained at 36.5 ± 0.5°C throughout all experiments. During the MRI scan, the rats were held in place fixed by a handmade ear bar and anesthetized through a facemask with 2% isoflurane (Saito et al., 2018). Transaxial 3-dimensional (3D) T_1_-weighted MR images (T_1_WIs) parameters were as follows: TR/TE = 400/9.57 ms, matrix = 256 × 224 × 45, field of view = 25.6 × 22.4 × 9.0 mm, average = 4, and spatial resolution = 100 × 100 × 200 μm. Osmotic pressure-controlled MnCl_2_ (0.002 ml/g, Sigma-Aldrich) was given by infusing 75 ml/kg of a 50 mM MnCl_2_ solution at a rate of 0.4 ml/h through the tail vein 24 h before MRI scanning.

After MRI scanning, all rats were used for histology in order to clarify the source of T_1_-changes by MEMRI in the hippocampus. All rats were euthanized by an overdose of pentobarbital (Dainippon Sumitomo Pharma Co., Ltd., Japan) and were prepared for histology by trans-cardiac perfusion with saline containing heparin followed by 4% paraformaldehyde. Removed brains were embedded in paraffin and coronal slices of 40 μm thickness corresponding to the coronal diagram (Bregma −3.12 mm) and orientations on the MR images were cut with reference to the Paxinos rat brain atlas (Paxinos and Watson, 1998). Slides were processed for Haematoxylin and Eosin (HE) staining and immunostaining for, neural cell adhesion molecule (NCAM), glial fibrillary acidic protein (GFAP); maker for astrocytes and ionized calcium binding adaptor molecule 1 (Iba1; marker for microglia). All staining procedures were performed on all 10 brains.

The hippocampus of the normal and radiation-exposed rats was subdivided into four regions: cornu ammonis region 1 (CA1), cornu ammonis region 2 (CA2), cornu ammonis region 3 (CA3), and dentate gyrus (DG). HE staining was performed to identify the cell density and assess alterations in microstructures, i.e., laminar structures. Distribution of hippocampal mossy fiber bundles was assessed using NCAM immunostaining (Sakata-Haga et al., 2003). Areas containing positive NCAM, GFAP and Iba1 staining were identified in the hippocampal sub-region of the immunohistochemical images as follows: (1) brightness and contrast were optimized automatically using Photoshop (Ver. 8.0.1, Adobe Inc., San Jose, CA, USA); (2) blue-stained intact cells were removed using the “color replacement tool” (allowance value = 200, Photoshop); (3) the color image was converted to a black-and-white binary map using the “make binary tool” (Ver.1.40g, National Institutes of Health, Bethesda, MD, USA); and (4) the size of the positive cell area was determined. The ratio of positive cell area to total area was calculated.

Region of interests (ROIs) were defined in the whole hippocampus and whole brain areas were manually selected. The most anterior hippocampus slice corresponded to a level of approximately 1.80 mm posterior to the Bregma and an orientation similar to the MR images with reference to the Paxinos rat brain atlas (Paxinos and Watson, 1998). The most posterior hippocampus slices corresponded to a level of approximately 5.0 mm posterior to Bregma. The calculated areas were greater than 3.2 mm thick. Approximately 16 hippocampus slices were identified in each animal. The enhanced hippocampus volume was defined by threshold at mean + 2 SD of the signal intensity of a ROI in the caudate putamen.

## Statistical Analysis

All statistical data are presented as mean ± standard deviation. All statistical analyses were performed using Prism7 (Version 7, GraphPad Software, CA, USA) and were focused on the differences of volume between the normal and radiation-exposed groups. An unpaired *t*-test was applied in order to compare volume changes as seen with MEMRI across groups of animals. MRVision (LASystems, Japan) was used for calculation, display and measurement of all of the images. MRI data and immunohistochemical staining were compared between groups using an unpaired *t*-test. Statistical significance was set at *p* < 0.05.

## Results

**Figure 1:**
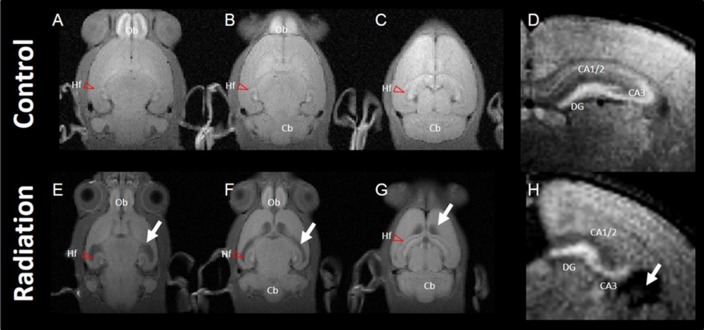
Typical images of manganese-enhanced MRI (MEMRI). Top panel: MEMRI of normal rat whole brain (**A–C**; horizontal slice) and hippocampus (**D**; axial slice). Bottom panel: MEMRI of a radiation-exposed rat (**E–G**; horizontal slice) and hippocampus (**H**; axial slice). White arrows indicate ventricle dilatation in **(E–H)**. T_1_ enhancement is seen around the whole hippocampal area of normal rat brain; however, T_1_ enhancement was different between normal and radiation-exposed rat brains, especially in dentate gyrus (DG) and CA3. Cb, cerebellum; Hf, hippocampal formation (red arrow head); Ob, olfactory bulb.

Axial images of MEMRI of the brain in control and radiation-exposed rats are shown in Figure 1. Signal intensities of MEMRIs were enhanced apparently in the olfactory bulb (Ob) and Hf in control rats (Figures 1A–D). In particular, MEMRI signals were enhanced strongly in the hilus and granular layer of the DG and the pyramidal layer and infrapyramidal region of the CA3, and moderately in the pyramidal layer of CA1 to CA2 (Figure 1D). In radiation-exposed rats, manganese was accumulated in brain regions similar to those in control rats. However, MEMRI signals in the Ob and Hf were decreased as compared to control rats. In the hippocampus of radiation-exposed rats, MEMRI signals sustained in the DG and CA3 regions, but disappeared in the CA1/2 (Figure 1H). Ectopic cell mass invading into the CA1/2 as seen by HE-stained sections was ambiguous by MEMRI. All radiation-exposed rats showed prominent dilatation of lateral ventricles with thinning the cerebral cortex (large white allows in Figures 1E–H).

**Figure 2:**
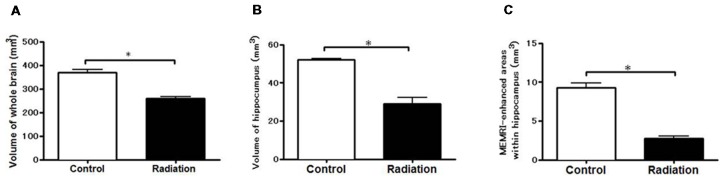
Volume-metric results of MEMRI. Volume-metrics were performed using MEMRI. **(A)** Whole-brain volumes from –7.2 mm to 2.0 mm using the Bregma as a reference. **(B)** Hippocampal volume at the same position. **(C)** Volume of the hippocampal region appearing MEMRI signal enhancements (at the same position). Significant lower volumes in radiation-exposed rats than in control rats were obtained either in the whole brain, hippocampus, or MEMRI signal-enhanced areas (*p < 0.001; unpaired *t*-test).

**Figure 3:**
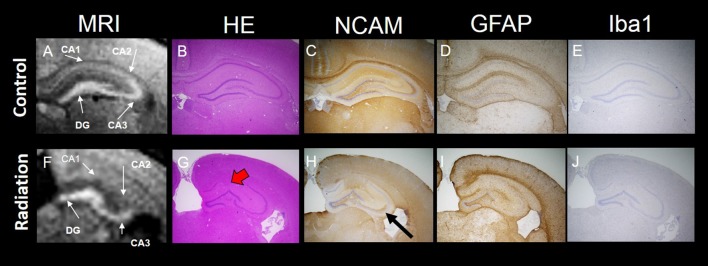
MEMRI and histology of the hippocampus. Top panel: MEMRI and histology of normal rat brain **(A–E)**. Bottom panel: MEMRI and histology of radiation-exposed rat **(F–J)**. The cell layers in the hippocampus were subdivided into four groups: CA1, CA2, CA3 and DG. **(A)** MEMRI of a normal rat hippocampal region. **(A,F)** MEMRI of a hippocampal region. **(B,G)** Haematoxylin and Eosin (HE) staining. **(C,H)** Neural cell adhesion molecule (NCAM) staining. **(D,I)** Glial fibrillary acidic protein (GFAP) staining. **(E,J)** Ionized calcium binding adaptor molecule 1 (Iba1) staining. HE staining was more intense in normal rats than in radiation-exposed rats **(B,G)**. Ectopic neurons were observed in radiation-exposed rats around CA1-2 (**G**, red arrow). NCAM immunoreactivity along the entire mossy fiber trajectory of CA3 in control rats was increased as compared to radiation-exposed rats (**C,H**, black arrow). The differences in positive staining were confirmed at GFAP and Iba1 only in DG between control and radiation-exposed rats **(D,I)**. Magnification: ×40; black bar at right side in **(E,J)** represents 300 μm.

**Figure 4:**
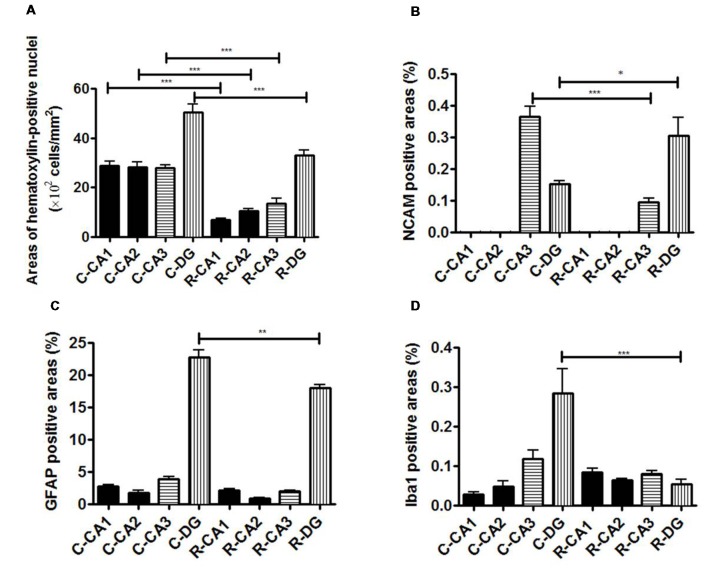
Evaluation of immunohistochemical staining. **(A)** Comparison of the ratio of the number of HE-positive cells in the hippocampal sub-regions. **(B)** Comparison of the ratio of NCAM-positive cell areas in the hippocampal sub-regions. **(C)** Comparison of the ratio of GFAP-positive cell areas in the hippocampal sub-regions. **(D)** Comparison of the ratio of Iba-1-positive cell areas in the hippocampal sub-regions. Significant difference in the cell density in CA1, CA2, CA3 and DG was detected between 3-week-old normal and radiation-exposed rat brains. Significant difference in the percentage area of NCAM-positive mossy fibers through the DG to CA3 was detected between 3-week-old normal and radiation-exposed rat brains. In all subregions of the hippocampus, there were significant decreases in the cell density and mossy fiber areas in radiation-exposed rats as compared to control rats. The density of GFAP-positive cells in DG was higher in control rats than in radiation-exposed rats (**C**; ***p < 0.001). In addition, there were differences in the distribution of Iba1-positive cells in DG between the control and radiation-exposed rats (**D**; ***p < 0.001). *p < 0.05; **p < 0.001; ***p < 0.0001; Tukey’s multiple comparison test. N-CA1-3, CA1-3 of a normal rat; N-DG, dentate gyrus of a normal rat; R-CA1-3, CA1-3 of a radiation-exposed rat; R-DG, dentate gyrus of a radiation-exposed rat.

Whole brain volume was significantly decreased in the x-ray-exposed rats as compared to control rats (Figure 2A; *p < 0.001). Radiation-exposed rats had smaller hippocampal volumes than control rats (Figure 2B; *p < 0.001). In addition, there was a significant difference between radiation-exposed and control rats in MEMRI-enhanced areas within the hippocampus (Figure 2C; *p < 0.001). By HE staining, hematoxylin-stained nuclei aligned in the DG granule cell layer and the CA3 pyramidal cell layer were deranged with a lower density in radiation-exposed rats (Figures 3B,G). Ectopic cell mass, which was known to appear typically with high doses of prenatal irradiation (Aoki et al., 1999), were invaded into CA1/2, destroying their laminar organization including the pyramidal cell layer (Figure 3G, red arrow). The density of hematoxylin-stained nuclei was significantly lower in radiation-exposed rats as compared to control rats in CA1 through CA3 and DG (Figure 4A; ***p < 0.001). Next NCAM immunostaining was carried out to evaluate mossy fiber distributions in the hippocampus of radiation-exposed rats. NCAM-positive mossy fibers were aberrantly distributed in the suprapyramidal region of the CA3 in radiation-exposed rats with significantly decreasing NCAM-positive areas (Figure 4B; *p < 0.05, **p < 0.01, ***p < 0.001).

MEMRI-enhanced signals in control rats were seen, corresponding to hippocampal regions that distributed densely hematoxylin-stained nuclei and/or NCAM-positive mossy fiber boundless (Figures 3A,C). This was reinforced by reducing MEMRI signals in the hippocampal regions corresponding to disorganized pyramidal layers and/or aberrant mossy fiber distribution (Figures 3A–C,F–H). We further evaluated the involvement of glial components in MEMRI signal enhancements. GFAP- and Iba1-positive areas, which reflects densities of astrocytes and microglia, were larger in the DG rather than in other hippocampal regions both in radiation-exposed and control rats. In both groups, astrocytes, rather than microglia, were dominant glial components in the DG, because Iba1-positive areas were less than one twentieth of GFAP-positive area within the DG of either radiation-exposed or control rat. As intergroup differences, GFAP-positive areas in the DG were lower in radiation-exposed rats as compared to controls (Figures 3D,I, 4C, ***p < 0.001). A significantly decreased Iba1-positive areas were also seen in the DG of radiation-exposed rats (Figures 3E,J, 4D). Thus, glial components such as astrocytes and microglia were densely presented in the DG, which appeared most intense signals of MEMRI both in radiation-exposed and control rats. However, the alteration of decreased density of glial components in MEMRI signal enchantments is small. The predominant glial components, astrocyte, were persisted to large populations in radiation-exposed rats albeit decreasing their density significant.

## Discussion

The present study demonstrated that *in vivo* MEMRI can detect and characterize prenatal irradiation-induced microstructural changes in the hippocampus as well as gross structural changes. The key findings were that: (1) signal enhancement of MEMRI could delineate different neuronal and/or glial components among hippocampal regions; and (2) reduced signal intensities in MEMRI agreed well with decreasing densities of histologically- and immunohistochemically-defined neurons and glial cells in the deformed hippocampus of prenatally radiation-exposed rats.

A significant lower brain weight was obtained reportedly in young adult rats with prenatal x-ray irradiation than in age-matched control rats (Miki et al., 1995). Consistently, at 3 weeks after birth, the brain volume of our radiation-exposed rats was significantly smaller as compared to control rats, as shown by the present MEMRI-based volumetric study. The present study further revealed a reduction in the hippocampal volume of radiation-exposed rats. In addition, the present study showed prominent dilatation of the lateral ventricles with thinning the cortex in radiation-exposed rats. The lateral ventricular dilatation may be a secondarily for prenatal irradiation-induced cerebral deformations such as the hippocampal hypoplasia and the cortical thinning. Thus, MEMRI-based qualitative and quantitative analyses can allow clarifying gross structural changes in the cerebrum involving in hippocampal deformations.

In the present study, the cell density decreased in all sub-regions of hippocampus as evaluated using HE staining in radiation-exposed rats. Miki et al. (1995) reported that prenatal radiation exposure caused a decreased number of neurons in the cerebral cortex with the cortical thinning in rats, albeit no altering in the numerical densities of neurons (Miki et al., 1995). Furthermore, a dose-dependent decrease of the cerebellar volume (Sawada et al., 2013) and a reduced number of total Purkinje cells (Li et al., 2002) were reported as the effect of prenatal radiation exposure on the rat cerebellum. Thus, the present findings of cell density alterations were not specific to the hippocampus. Neuronal loss by prenatal irradiation may be a region-nonspecific, and involved in the brain hypoplasia as seen in the present study and others (Miki et al., 1995).

MEMRI signal enhancements and histological alterations such as HE staining and immunostaining for NCAM, GFAP and Iba1 were compared in order to investigate the mechanism of signal alterations of MEMRI in prenatal radiation-exposed rats. In radiation-exposed rats, MEMRI signals were obtained in hippocampal regions identical to the DG through CA3 regions, including infra- and suprapyramidal regions as well as the granular and pyramidal layers. In parallel, the hippocampal cell density decreased in all sub-regions in radiation-exposed rats as evaluated using HE staining. In particular, MEMRI signals disappeared in the CA1/2 regions, corresponding to the regions that the pyramidal layer was deranged by invading ectopic cell mass, resulting in an interspersion of neurons. T_1_ relaxation enhancement by Mn^2+^ is proportional to the ion concentration taken into the cells through Ca^2+^ channels [approximately (Mn) <1 mM]; thus, the cell density is the one source of MEMRI signal changes (Silva et al., 2004). This suggests that reduced MEMRI signals in the radiation-exposed rat hippocampus are reflected by decreasing the cell density However, the ectopic cell mass was undistinguishable by MEMRI so that we detected deformations of the CA1/2 in prenatal radiation-exposed rats as the pyramidal layer derangement by disappearing MEMRI signal enhancements. Mossy fibers in the hippocampus project from the DG to CA3. The pathway consists of varicose granule cell axons that terminate on the dendrites of hilar mossy cells and pyramidal cells in CA3. No mossy fibers were distributed in the CA1 subregion. The most pronounced enhancement in MEMRI was detected in CA3 and DG subregions matching the mossy fiber pathways in previous reports (Watanabe et al., 2004; Nairismägi et al., 2006). Moreover, MEMRI sustained in the DG granular layer and CA3 pyramidal layer, even in prenatal radiation-exposed rats, although the cell density in those regions were reduced to about half of controls. NCAM-positive mossy fiber bundles were retained in those regions as aberrant infrapyramidal fibers in radiation-exposed rats, while NCAM-stained areas were increased in the DG, but slightly decreased in the CA3. MEMRI after systemic injections of MnCl_2_ has been shown to detect axonal and mossy fiber sprouting in the hippocampus following kainite-induced status epilepticus (Immonen et al., 2008). The present findings suggest that an accumulation of manganese in the hippocampal mossy fibers is involved in sustained MEMRI signals in the DG and CA3 regions of radiation-exposed rats.

Recently, some studies have demonstrated that MEMRI is a useful technique for the detection of brain activity and cytoarchitectonic details of the hippocampal area at a cellular level (Aoki et al., 2004; Nairismägi et al., 2006; Immonen et al., 2008). The mechanism of MEMRI signal enhancement has been previously discussed in relation to experimental models of hypoxia (Wideroe et al., 2007). Ischemic models result in MEMRI signal enhancement as a reaction of gliosis (Kawai et al., 2005, 2007). The present immunohistochemical results revealed that densities of astrocytes and microglia were decreased in the DG of radiation-exposed rats. However, the alteration of reduced density of glial components in MEMRI signal enchantments may be minimum. The predominant glial components, astrocyte, were persisted to large populations in radiation-exposed rats albeit decreasing their density significantly. Therefore, the non-uniform distribution of glial components within the hippocampus may also contribute to sustaining strong MEMRI signals in the DG of prenatal radiation-exposed rats, as well as mossy fiber bundles.

## Conclusion

The present study using a prenatal radiation-exposed rat model revealed that MEMRI signal enhancements in the hippocampus were associated with densities of neuronal and glial components. In particular, MEMRI signal enhancements delineated different neuronal and/or glial components among hippocampal regions: the pyramidal cell layer of the CA1/2 regions; the pyramidal cell layer and infrapyramidal/suprapyramidal mossy fiber bundles in the CA3; the DG granular layer; and mossy fiber bundles and astrocytes in the DG hilus. Therefore, the present *in vivo* MEMRI findings provide advantageous information for detecting nondestructively hippocampal deformations in neurodevelopmental disorders.

## Author Contributions

SS and IA designed the study, and wrote the initial draft of the manuscript. SS and KS contributed to analysis and interpretation of data, and assisted in the preparation of the manuscript. All authors have contributed to data collection and interpretation, and critically reviewed the manuscript. All authors approved the final version of the manuscript, and agree to be accountable for all aspects of the work in ensuring that questions related to the accuracy or integrity of any part of the work are appropriately investigated and resolved.

## Conflict of Interest Statement

The authors declare that the research was conducted in the absence of any commercial or financial relationships that could be construed as a potential conflict of interest.
